# Association of decreased grip strength with lower urinary tract symptoms in women: a cross‐sectional study from Korea

**DOI:** 10.1186/s12905-021-01241-4

**Published:** 2021-03-04

**Authors:** Su-Jin Yang, Jung Ha Park, Yunhwan Oh, Hyeonju Kim, Mihee Kong, Jihyun Moon

**Affiliations:** 1 School of Medicine, Jeju National University, Jeju, Republic of Korea; 2 Department of Family Medicine, Jeju National University Hospital, Aran 13gil 15, Jeju-si, Jeju, 690-767 Republic of Korea; 3 Department of Family Medicine, School of Medicine, Jeju National University, Jeju, Republic of Korea

**Keywords:** Muscle strength, Overactive bladder, Urinary incontinence

## Abstract

**Background:**

Lower urinary tract symptoms (LUTS) including frequency, nocturia, urgency, and incontinence, are common in women and cause significant discomfort in daily life. However, diagnosis and treatment of LUTS are often delayed because many patients with such symptoms do not complain to the physician of discomfort and do not seek medical attention. LUTS are known to be associated with muscle weakness. We investigated the association between grip strength and LUTS in women of different ages.

**Methods:**

This study included 4225 women (mean age 48.6 years) who underwent self-referred health screening between April 2015 and December 2019. LUTS were evaluated using a self-reported questionnaire, and the overactive bladder symptom score was used to screen for an overactive bladder. Low muscle strength was defined as a hand grip strength of < 18 kg (decreased grip strength).

**Results:**

We observed decreased grip strength in 13.7% (n = 580) of the participants. Nocturia, urgency, incontinence, and overactive bladder were more common in women with decreased grip strength than in women with normal grip strength. After adjusting for age, comorbidities (hypertension, diabetes, hyperlipidemia), smoking status, alcohol consumption, regular exercise, and stress, nocturia (odds ratio [OR] 1.19, 95% confidence interval [CI] 1.01–1.52), urinary incontinence (OR 1.32, 95% CI 1.01–1.72), and an overactive bladder (OR 1.75, 95% CI 1.35–2.27) were significantly associated with decreased grip strength.

**Conclusions:**

The findings suggest that LUTS, especially nocturia, incontinence, and an overactive bladder are associated with decreased grip strength in women. Therefore, physicians should be aware that patients may not seek help, even if they are uncomfortable, and it is important to obtain a detailed medical history and perform additional tests, even in the absence of complaints, in patients with low grip strength, who are at high risk of LUTS.

**Supplementary Information:**

The online version contains supplementary material available at 10.1186/s12905-021-01241-4.

## Background

Lower urinary tract symptoms (LUTS) are common in women of all ages worldwide, although the prevalence of symptoms differs across various study groups. For example, the prevalence of overactive bladder (OAB) is 19.7% in South Korea [1], and that of urinary incontinence (UI) is in the range of 25–45% [2]. LUTS can impair one’s quality of life and increase the risk of falls and fractures [3]. Furthermore, LUTS are related to sleep disorders, depression, and anxiety [4]. One Italian study showed that OAB is associated with increased medical costs, and the total cost of diagnosis, treatment, and care for adverse outcomes related to OAB are more than $9 billion [5].

Diagnosing urologic diseases starts with obtaining a medical history and continues with a urinary diary or questionnaires, a physical examination, and tests. The most important aspect of the diagnosis is identifying symptoms since the majority of patients do not report their discomfort [6]. In a previous study, less than half of the studied patients with symptoms of UI actually visited a physician [7]. Low help-seeking behavior results in a delayed diagnosis and an extended treatment period. Therefore, for patients who are at a high risk of developing LUTS, it is important to obtain their detailed medical history and conduct further examinations before they complain about their symptoms.

Aging is also related to LUTS, as it is related to a decreased bladder capacity, decreased ability to postpone voiding, and deregulation of bladder function due to reduced muscular and neurologic activities [8]. For this reason, urinary disorders are common in older women, and physicians are aware of this issue, but in reality, these symptoms are experienced by young adults as well as older people [9]. However, young adults tend to delay or avoid seeking medical care [10], which further delays their treatment. Thus, it is crucial to identify patients, especially younger women, before their symptoms deteriorate.

Recently, many studies have examined the correlation between body composition and LUTS. Sarcopenia (a decline in skeletal muscle mass and strength and/or reduced physical performance) [11] is associated with various LUTS in both men and women [12]. A study reported significant correlations between sarcopenia and OAB [13], and one study in Europe suggested that stress urinary incontinence (SUI) might be caused by loss of sphincter muscle [14]. Likewise, another study showed that atrophy and/or weakness of the pelvic floor muscles may result in changes in the pelvic musculature and thereby UI [14]. Furthermore, the symptoms of OAB or SUI can be improved through exercises such as pelvic muscle training [15]. However, the diagnosis of sarcopenia, which involves measuring appendicular skeletal muscle mass, is difficult to perform in a clinical setting. Moreover, it is difficult to assess pelvic muscle strength. Therefore, a marker that highly correlates with body composition is needed, and grip strength is one such marker that is relatively easy to measure and significantly correlated with muscle mass and strength. Grip strength has also been reported to predict CVD [16], cancer [17], and is closely linked to mortality [18]. However, a study that investigated the association of muscle thickness of transverse abdominal muscle and pelvic floor with UI in women reported that those with UI had thinner abdominal and pelvic floor muscles than those without UI [19]. However, grip strength scores did not significantly differ between the groups. Therefore, it is still debatable to what extent grip strength can reflect the quantity and quality of pelvic floor muscles.

In this study, we assumed that LUTS occur in relation to muscular changes. Thus, we aimed to determine the relationship between grip strength and LUTS such as daytime urinary frequency, nocturia, urgency, and incontinence.

## Methods

### Study design and participants

This retrospective, cross-sectional study included women aged over 19 years (average age 48.6 years) who underwent a self-referred health screening. The screening involved the completion of self-reported questionnaires, taking physical measurements, and analyzing laboratory tests at Jeju National University Hospital from April 2015 to January 2019. The questionnaire developed for this study is provided as Additional file 1. Participants with active malignancy who were receiving therapeutic treatment were excluded from this study. Thus, a total of 4225 women were included in this study.

## Grip strength

Grip strength was measured using a hand dynamometer (Jamar 5030J1; Sammons Preston, Bolingbrook, IL, USA). Each participant’s grip strength was measured twice for both hands alternately. Under the supervision of trained inspectors, participants squeezed the dynamometer with full force for at least three seconds, followed by a rest time of less than 30 s. The higher grip score was used for the statistical analysis. Based on the consensus of Asian Working Group for Sarcopenia, < 18 kg was considered decreased grip strength [11].

## Lower urinary tract symptoms

Self-reported questionnaires were used to assess LUTS, particularly storage symptoms and OAB, based on which the OAB symptoms score (OABSS) was obtained. The Korean version of the OABSS has been translated and linguistically validated in a previous study [20]. This self-administered questionnaire consists of four categories: frequency during the day, frequency during the night, urgency, and UI. Frequency of more than eight times during the day was defined as increased daytime frequency [21]; nocturia was defined as waking up at night one or more times to urinate [22]. Any involuntary leakage of urine was considered UI [22]. Participants with an urgency score of two or more and an OABSS of three or more were considered to have OAB [23]. A total score of five or less was classified as mild OAB, 6–11 was classified as moderate OAB, and 12 or more was classified as severe OAB.

## Covariates

Data on age, medical history, comorbidities with current medication, smoking status, drinking status, physical activity level, and stress level assessed by the Korean-translated Brief Encounter Psychosocial Instrument (BEPSI-K) [24] were acquired through self-reported questionnaires. Participants’ height, weight, and waist circumstance were measured by trained nurses. Body fat percent was measured using a bioelectrical impedance analysis machine (InBody 720; InBody, Seoul, Korea). Further, following an overnight fast of 10 h, laboratory tests, including a complete blood count and for serum albumin, uric acid, high-sensitivity C-reactive protein assay, thyroid-stimulating hormone, and 25-hydroxyvitamin D, were conducted. A Homeostatic Model Assessment for Insulin Resistance (HOMA-IR) was also performed.

### Statistical analysis

Participant baseline characteristics are presented as either means ± standard deviations (SD) or numbers (%). We performed an analysis of covariance to verify the relationships of grip strength with daytime frequency, nocturia, incontinence, and OAB. The association between grip strength and LUTS (nocturia, incontinence, and OAB) was assessed by multiple logistic regression analysis, after adjusting for age, hypertension, diabetes, dyslipidemia, smoking status, alcohol consumption, exercise, and stress level. SPSS version 23.0 software program (IBM Corp., Armonk, NY, USA) was used for statistical analysis, and a *p* value < 0.05 was considered to determine statistical significance.

## Results

### Participants’ general characteristics

Of the total participants, 13.7% showed decreased grip strength (< 18 kg). Women with decreased grip strength were significantly older (*P* < 0.001) and had higher BMI (*P* = 0.016) than did women with intact grip strength. Comorbidities such as hypertension (*P* < 0.001), diabetes (*P* < 0.001), dyslipidemia (*P* = 0.002), smoking status (*P* = 0.010), exercise (*P* = 0.012), and level of stress (*P* = 0.001) were significantly different between groups (Table 1).

**Table 1:** Participants’ baseline characteristics by grip strength

Grip strength	Normal (N = 3645)	Impaired (N = 580)	*p*
Age, years	47.6 ± 10.5	54.7 ± 13.0	< 0.001
Height, cm	158.1 ± 5.4	154.7 ± 5.8	< 0.001
Weight, kg	59.1 ± 8.6	57.4 ± 8.0	< 0.001
Body mass index, kg/m^2^	23.7 ± 3.3	24.0 ± 3.3	0.016
Waist circumference, cm	79.8 ± 8.5	81.3 ± 8.7	< 0.001
Body fat, %	33.0 ± 6.0	35.1 ± 6.2	< 0.001
Albumin, g/dL	4.2 ± 0.2	4.1 ± 0.2	0.005
Uric acid, mg/dL	4.6 ± 1.0	4.7 ± 1.0	0.002
HbA1C, %	5.5 ± 0.6	5.7 ± 0.7	< 0.001
HOMA-IR	1.6 ± 1.2	1.7 ± 1.8	0.037
TSH, µIU/mL	2.2 ± 1.7	2.1 ± 1.3	0.020
25-OH-vitamin D, ng/mL	19.9 ± 10.4	20.9 ± 10.8	0.054
Hypertension	339 (9.3)	92 (15.9)	< 0.001
Diabetes	89 (2.4)	34 (5.9)	< 0.001
Dyslipidemia	270 (7.4)	65 (11.2)	0.002
Current smoker	136 (3.7)	37 (6.4)	0.010
Alcohol consumption	481 (13.2)	59 (10.2)	0.050
Regular exercise	826 (22.7)	104 (17.9%)	0.012
Stress^a^			0.001
Low	1897 (52.0)	287 (49.5)	
Moderate	1457 (40.0)	220 (37.9)	
High	291 (8.0)	73 (12.6)	
Daytime frequency	1106 (30.3)	194 (33.4)	0.145
Nocturia	1935 (53.1)	378 (65.2)	< 0.001
Urgency	320 (8.8)	109 (18.8)	< 0.001
Incontinence	329 (9.0)	93 (16.0)	< 0.001
Overactive bladder			< 0.001
None	3339 (91.6)	473 (81.6)	
Mild	169 (4.6)	48 (8.3)	
Moderate	131 (3.6)	54 (9.3)	
Severe	6 (0.2)	5 (0.9)	

Data are presented as mean ± standard deviation or number (%)

Values were calculated using T-test and Chi square test

^a^Stress status was defined using the Brief Encounter Psychosocial Instrument-Korean version score

### **Association between grip strength and** LUTS

**Fig. 1:**
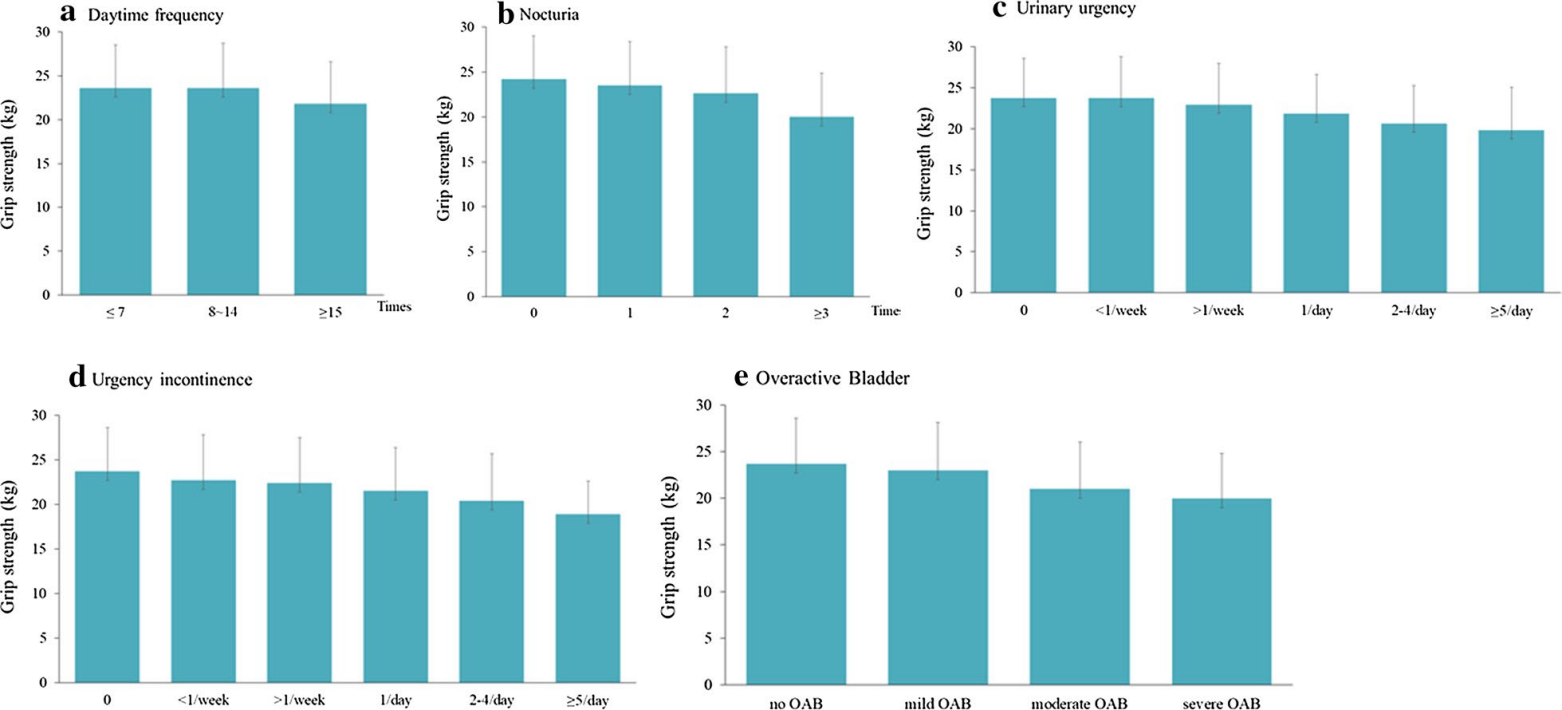
Age-adjusted mean of grip strength in women with LUTS. Values were calculated using one-way analysis of variance and the Jonckheere–Terpstra test

Nocturia, urgency, and incontinence were more common in the decreased grip strength group than in the intact grip strength group (*P* < 0.001). Similarly, decreased grip strength was associated with OAB (*P* < 0.001). However, daytime frequency did not differ between the two groups (*P* = 0.145) (Table 1). Grip strength was significantly associated with frequency of nocturia, urgency, and incontinence, as well as with OAB severity (all *Ps* for trend < 0.001). Mean (SD) grip strength scores for participants with nocturia frequencies of 0, 1, 2, and over 3 times per night were 24.2 (4.8), 23.5 (4.9), 22.6 (5.2), and 20.0 (4.9), respectively. In case of UI, women without leakage had the highest grip strength (23.7 ± 4.9). Conversely, the mean (SD) grip strength score was 22.7 (5.1) for those who reported leakage less than once a week, 22.4 (4.9) more than once a week, 21.5 (4.3) once a day; the lowest score was 18.9 (3.7) in those who reported leakage more than five times a day. Similar patterns were observed with respect to grip strength and OAB severity. The mean (SD) grip strength score of participants without OAB was 23.7 (4.9), 23.0 (5.1) in participants with mild OAB, 21.0 (5.0) in participants with moderate OAB, and 20.0 (4.8) in participants with severe OAB (Fig. 1).

In the multivariable-adjusted logistic regression analyses after adjusting for age, comorbidities (hypertension, diabetes, dyslipidemia), lifestyle factors (smoking status, alcohol consumption, regular exercise, and high stress), participants with decreased grip strength had 1.19 times higher odds of nocturia (95% CI 1.01–1.52), 1.32 times higher odds of incontinence (95% CI 1.01–1.72), and 1.75 times higher odds of OAB (95% CI 1.35–2.27) (Table 2).

**Table 2:** Factors associated with nocturia, incontinence, and OAB by multiple logistic regression

	Nocturia		Urinary incontinence		Overactive bladder	
	OR	95% CI	OR	95% CI	OR	95% CI
Grip strength	1.19	(1.01–1.52)	1.32	(1.01–1.72)	1.75	(1.35–2.27)
Age	1.09	(1.08–1.10)	1.04	(1.03–1.05)	1.03	(1.02–1.04)
Hypertension	1.45	(1.11–1.89)	1.12	(0.8201.54)	1.48	(1.08–2.03)
Diabetes	1.84	(1.19–2.84)	1.83	(1.15–2.92)	1.61	(0.98–2.63)
Dyslipidemia	1.16	(0.86–1.57)	1.35	(0.96–1.91)	1.06	(0.74–1.53)
Current smoker	1.22	(0.98–1.51)	1.08	(0.85–1.37)	1.11	(0.88–1.41)
Alcohol consumption	1.12	(0.82–1.53)	0.94	(0.67–1.33)	0.88	(0.62–1.24)
Regular exercise	0.83	(0.66–1.05)	0.81	(0.62–1.05)	0.88	(0.67–1.14)
High stress	1.67	(1.44–1.93)	1.58	(1.36–1.85)	1.83	(1.57–2.14)

Odds ratios (95% confidence intervals) were calculated by multivariable logistic regression analysis

*OR* odds ratio, *CI* confidence interval

## Discussion

In this study, we found that LUTS were associated with decreased grip strength in women. Furthermore, the likelihood of OAB was also higher in women with decreased grip strength.

The results of this study are similar to those of previous studies that examined the correlation between grip strength and LUTS. In a study on 858 older women in Japan, participants with nocturia frequency of 2–4 times per night had lower grip strength scores than did those with nocturia frequency of 0–1 times per night [25]. Another study on 1399 women aged 75–84 years investigated the relationship between musculoskeletal condition and UI by examining body composition and physical functions such as grip strength. They found that increased grip strength was related to reduced likelihood of UI (OR = 0.946, 95% CI = 0.912–0.981) [26]. In a cohort-study on participants aged 70–79 years, those with more than 5% decrease in grip strength over 3 years were 1.6 times more likely to have a new diagnosis of SUI [27].

Sarcopenia and LUTS are associated with each other, especially in the case of UI. Women with sarcopenia, adjusted by weight, have a 1.5 times higher rate of SUI than do typical women [14]. Pelvic floor muscles are composed of levator ani muscle, endopelvic fascia, and supporting ligaments, and these have important roles in supporting lower urinary structures, Pelvic floor muscles form a “hammock” that provides support under the urethra [28]. Decline in the mass of these muscles linked with sarcopenia affects urinary functions. In other words, decrease in appendicular skeletal muscle mass is accompanied by a decrease in mass and strength of muscles that support the lower urinary tract. Weak pelvic floor muscles can cause UI because the urethra is unable to resist the rising bladder pressure [29]. Another study found that sarcopenia causes inflammatory cytokines and oxidative stress, which in turn can cause myopathy and neurodegeneration of bladder, and thereby OAB. Another recent study suggested a possible correlation between the development of OAB and bladder vascular insufficiency or bladder ischemia [30].

Sarcopenia is accompanied by weakened muscles and the degeneration of functions due to decreased limb muscle mass. Measuring grip strength is relatively simple and quick, and it can allow patients to self-monitor. Women with decreased grip strength, as seen in our study, are more likely to have sarcopenia and thus experience various LUTS. We could predict sarcopenia in patients who visit primary care by measuring grip strength and make an early diagnosis by screening groups at high-risk for LUTS through detailed history-taking. Further large-scale epidemiological studies including participants of multiple races are still needed to determine the elaborate association between muscle mass, strength, and LUTS.

Further, a number of studies have reported the reasons for patients with LUTS not seeking treatment [31]; one is that these symptoms are considered trivial or not problematic enough, irrespective of objective severity. Another reason is that many individuals think that their LUTS occur because of ageing or childbirth. Further, lack of knowledge of treatment options, embarrassment, and fear of invasive investigations also act as barriers to treatment-seeking behavior. Because of these reasons, it is difficult to make early diagnosis until patients complain about their symptoms. Therefore, various objective indicators, such as grip strength and medical history of high-risk groups, are crucial for early and accurate diagnosis and treatment of LUTS.

There are several limitations to this study. First, the study used a cross-sectional design, which precludes inference of a cause–effect relationship. Second, the sample consisted of only women. However, men often develop more LUTS than women due to prostate disease. With this in mind, the causes of LUTS in men may differ from those in women, which suggests the necessity of further investigation using only female samples. Lastly, the tests were not conducted in this study, except for the administration of questionnaires. However, symptoms reported by patients are the most important factor in the diagnosis of urologic diseases; therefore, further examinations can be carried out based on patients’ symptoms.

## Conclusions

Our findings suggest that grip strength is associated with LUTS. Moreover, decreased grip strength was observed in women with OAB. We recommend that physicians recognize that women with decreased grip strength may have LUTS even if they do not seek help, and women with low grip strength may require additional testing for urologic disease. Furthermore, we can speculate that preventing sarcopenia may also serve to prevent LUTS as a primary deterrence.

## Supplementary Information

**Additional file 1.** Health Check-up Questionnaire.

## Data Availability

The datasets used and/or analyzed during the current study are available from the corresponding author on reasonable request.

## Abbreviations

CBC: Complete blood count
LUTS: Lower urinary tract symptoms
OAB: Overactive bladder
SD: Standard deviations
SUI: Stress urinary incontinence
UI: Urinary incontinence
