# Supplementary material for: Association of decreased grip strength with lower urinary tract symptoms in women: a cross‐sectional study from Korea
Source: BMC Womens Health. 2021 Mar 4;21:96. doi: 10.1186/s12905-021-01241-4 (PMC7934376; doi:10.1186/s12905-021-01241-4)
Supplement: Supplementary file 1 — Additional file 1. Health Check-up Questionnaire. [file 12905_2021_1241_MOESM1_ESM.pdf]

Health Check-up Questionnaire

Name		Birthday		Sex/Age	/
Hospital ID		Check-up date			

DISEASE

1. Please indicate below if you have any current medical condition.

Hypertension		Diabetes		Hyperlipidemia	
Tuberculosis		Stroke		Heart Disease	
Liver Disease		Thyroid Disease		Osteoporosis	
Cancer		disease entity			
Etc. (if you have any other diagnosed disease)					

2. Please indicate below if any of your family members have current medical condition.

Hypertension		Diabetes		Hyperlipidemia	
Tuberculosis		Stroke		Heart Disease	
Liver Disease		Thyroid Disease		Osteoporosis	
Cancer		disease entity			
Etc. (if you have any other diagnosed disease)					

LIFE STYLE

1. Do you smoke?

☐ Never	
☐ No, only in the past	
☐ Yes	The amount of smoking per day<br>pack/day
	The period of smoking<br>year(s) month(s)

2. Do you drink?

☐ No	
☐ Yes	How often do you drink per week?<br>day/week
	How much do you usually drink at once?<br>☐ 1–2 glasses ☐ 3–4 glasses<br>☐ 1 bottle ☐ 2 bottles ☐ more than 3 bottles

3. Do you exercise?

☐ No	
☐ Yes (예)	How often do you exercise per week?<br>day/week

STRESS

BEPSI questionnaire

1. In the past month have you ever felt as if there are more demands in your life, emotionally and physically, than you can handle comfortably?	☐ always<br>☐ frequently<br>☐ sometimes<br>☐ rarely<br>☐ never
In the past month, have you ever felt frustrated trying to live up to your own expectations or standards?	☐ always<br>☐ frequently<br>☐ sometimes<br>☐ rarely<br>☐ never
3. In the past month, have you ever felt that your needs as a person are being left unmet?	☐ always<br>☐ frequently<br>☐ sometimes<br>☐ rarely<br>☐ never
4. In the past month have you ever felt uncertain or apprehensive about the future?	☐ always<br>☐ frequently<br>☐ sometimes<br>☐ rarely<br>☐ never
5. In the past month, have you ever felt that there are so many everyday hassles and crises that you lose track of the things that are really important to you?	☐ always<br>☐ frequently<br>☐ sometimes<br>☐ rarely<br>☐ never

OABSS

Please select the option that applies best to your urinary conditions during the last week.

1. How many times do you typically urinate from waking in the morning until sleeping at night?	☐ 7 or less<br>☐ 8 to 14<br>☐ 15 or more
2. How many times do you typically wake up to urinate from sleeping at night until waking in the morning?	☐ 0<br>☐ 1<br>☐ 2<br>☐ 3 or more
3. How often do you have a sudden desire to urinate, which is difficult to defer?	☐ not at All<br>☐ less than once a week<br>☐ once a week or more<br>☐ about once a day<br>☐ 2 to 4 times per day<br>☐ 5 times a day or more
4. How often do you leak urine, because you cannot defer the sudden desire to urinate?	☐ not at All<br>☐ less than once a week<br>☐ once a week or more<br>☐ about once a day<br>☐ 2 to 4 times per day<br>☐ 5 times a day or more
